# Real-World Experience of Safety of Mycophenolate Mofetil in 119 Japanese Patients with Systemic Lupus Erythematosus: A Retrospective Single-Center Study

**DOI:** 10.1155/2021/8630596

**Published:** 2021-01-23

**Authors:** Yoshiyuki Abe, Kurisu Tada, Ken Yamaji, Naoto Tamura

**Affiliations:** Department of Internal Medicine and Rheumatology, Juntendo University School of Medicine, Tokyo, Japan

## Abstract

**Objectives:**

Mycophenolate mofetil (MMF) is the standard treatment for lupus nephritis. In Japan, it was approved for lupus nephritis in 2015. We investigated its real-world safety and effectiveness in Japanese patients with systemic lupus erythematosus (SLE).

**Methods:**

We analyzed the continuation rate, adverse events, and reasons for discontinuation of MMF in Japanese patients with SLE in a retrospective single-center study. We included 119 patients who received MMF from 31 July 2015 to 31 May 2019. To compare demographic and clinical characteristics between groups, the Mann–Whitney *U*-test was used for nonnormally distributed variables. Categorical variables were compared using Fisher's exact test. Kaplan–Meier curves were plotted for the discontinuation rate of MMF.

**Results:**

Patients consisted of 18 males and 101 females. Thirty-five patients discontinued MMF. The cumulative discontinuation rate was 42.4%. Twenty-nine patients discontinued MMF due to adverse events, and six patients discontinued MMF due to remission of SLE or desire for childbearing. At the time of the last observation, the lupus low disease activity state achievement rate was significantly lower in patients who experienced adverse events than those who did not (64% vs. 35%, *P* = 0.009). We examined the concentration of mycophenolate acid (trough level) in stored frozen serum in 11 patients. Two patients had irreversible complications due to viral meningitis; their trough mycophenolate acid concentrations were 8.3 and 6.3 *μ*g/mL, respectively.

**Conclusions:**

Although MMF may be effective in Japanese patients with SLE, physicians should pay attention to infections in patients with high mycophenolate acid concentrations.

## 1. Introduction

Mycophenolate mofetil (MMF) is an immunosuppressant that was approved for the prevention of transplant rejection in 1992 [1]. MMF is an ester of mycophenolate acid (MPA), the active metabolite. Since MPA inhibits de novo purine synthesis, on which only lymphocytes depend, MPA has specific antiproliferative effects [2]. The MMF is used as an immunosuppressant to treat rheumatic diseases, especially lupus nephritis [3, 4]. The American College of Rheumatology (ACR) and European League Against Rheumatism (EULAR) published guidelines for lupus nephritis in 2012 in which they recommended that MMF and cyclophosphamide should be considered equivalent for patients with International Society of Nephrology class III/IV lupus nephritis [5–7]. MMF is currently an important treatment for systemic lupus erythematosus (SLE).

MMF often causes adverse events. Typical adverse events include infections, upper gastrointestinal symptoms, and diarrhea [5]. ACR guidelines recommend that “Asians compared to non-Asians might require lower doses of MMF for similar efficacy” [7–9]. Previous Japanese studies have reported MMF doses of almost 1,000–2,000 mg/day/person for lupus nephritis [10–14]. In this single-center retrospective study, we evaluated medication continuation, adverse events, and reasons for discontinuation of MMF in 119 patients with SLE.

## 2. Patients and Methods

### 2.1. Patients

This retrospective single-center observational study included 173 patients who received MMF treatment from 31 July 2015 to 31 May 2019 at Juntendo University Hospital. MMF was approved for SLE on 31 July 2015 in Japan. We ultimately analyzed 119 of the initial 173 patients. We excluded 54 patients because 8 patients started MMF before 31 July 2015, 20 patients had been treated with MMF at another hospital, and 26 patients were treated with MMF for other rheumatic diseases. All 119 patients were diagnosed with SLE according to the 1997 ACR SLE classification criteria [15]. There was no standardized treatment regimen in this retrospective observational study; MMF treatment was determined by each patient's physician. The ethics committee of Juntendo University Hospital approved this study (approval number 19-054). Patients could opt out of the study through the hospital's website.

### 2.2. Clinical Evaluations and Outcomes

Clinical data, including patient demographics, clinical manifestations, laboratory data, outcomes, adverse events, and reasons for MMF discontinuation, were obtained from medical records. We analyzed clinical manifestations from any point in time. Laboratory data were from the time of MMF initiation. Treatments included all therapies received by patients at the time of MMF initiation and history of treatments before starting MMF. The primary outcome was the rate of MMF continuation. Secondary outcomes were adverse events associated with MMF, reasons for MMF discontinuation, MMF dose during the last observation period, and prevalence of the lupus low disease activity state (LLDAS) during the last observation period [16]. In this study, a major SLE flare was defined as British Isles Lupus Assessment Group Index category A disease [17].

### 2.3. Measurement of Serum MPA Levels

Serum MPA levels were measured retrospectively with frozen serum. We stored surplus serum after clinical laboratory testing for retrospective testing (Juntendo University Hospital ethics committee approval number 334). All measurements were performed using the enzyme multiplied immunoassay technique on serum samples collected when the MMF dose had been stable for more than a week. In order to collect serum at precisely 12 hours after administration to assess trough levels, only serum samples from hospitalized patients with confirmed blood collection times were selected.

### 2.4. Statistical Analysis

To compare demographic and clinical characteristics between groups, the Mann–Whitney *U*-test was used for nonnormally distributed variables. Categorical variables were compared using Fisher's exact test. Spearman's correlation coefficients were calculated. Kaplan–Meier curves were plotted for the discontinuation rate of MMF. Data are presented as medians (interquartile range (IQR)). Analyses were performed using SPSS version 23.0 software (SPSS, Armonk, NY) with *P* < 0.05 considered to be statistically significant.

## 3. Results

**Figure 1:**
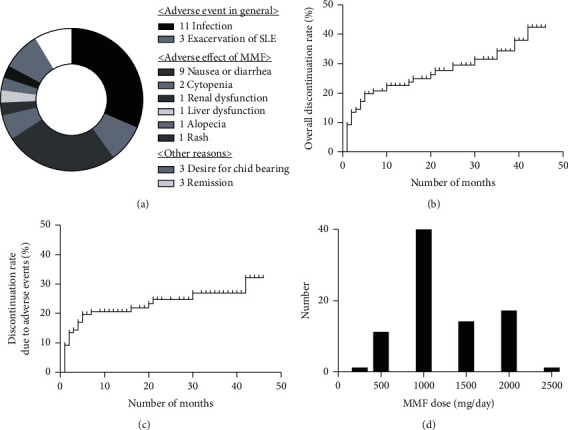
Details about MMF use. (a) Distribution of reasons for MMF discontinuation. (b) The Kaplan-Meier curve for MMF discontinuation. (c) The Kaplan-Meier curve for MMF discontinuation due to adverse events. (d) MMF dose at last observation. MMF: mycophenolate mofetil.

The median observation period was 16 (5–33) months. Patients consisted of 18 males and 101 females. The median age was 38 (31–46) years, and the median duration of SLE was 138 (39–242) months. Ninety-six patients received MMF as induction therapy and 23 patients received MMF as maintenance therapy. MMF was used for the following reasons: lupus nephritis (70 patients), serological abnormality (24 patients), change from another immunosuppressant (10 patients), rash (8 patients), neuropsychiatric manifestation (3 patients), cytopenia (2 patients), and arthritis (2 patients). Thirty-five patients discontinued MMF. The cumulative discontinuation rate was 42.4%. Twenty-nine of 35 patients discontinued MMF because of adverse events and six patients discontinued due to SLE remission or desire for childbearing. The reasons for discontinuation were as follows: infection (11 patients), nausea or diarrhea (9 patients), SLE exacerbation (3 patients), SLE remission (3 patients), desire for childbearing (3 patients), cytopenia (2 patients), and renal dysfunction, liver dysfunction, alopecia, and rash (1 patient each) (Figure 1(a)). For all adverse events, infection and SLE exacerbation were differentiated from general adverse events. Nausea or diarrhea, cytopenia, renal dysfunction, liver dysfunction, alopecia, and rash were distinguished from adverse effects of MMF.  Table 1 shows the background characteristics, laboratory test findings, treatments, and outcomes in all patients and by adverse event status. The group with adverse events had lower hemoglobin, as well as higher alanine aminotransferase (ALT) and blood urea nitrogen (BUN) levels. At the last observation, the LLDAS achievement rate was significantly lower in the adverse event group than in the no-adverse event group (64% vs. 35%; *P* = 0.009). Supplemental Table 1 shows the comparison between patients who received MMF as induction therapy versus maintenance therapy. The induction therapy group had lower concentrations of complement and higher anti-deoxyribonucleic acid (DNA) antibody titers and glucocorticoid (GC) dose at the start of MMF therapy. Supplemental Tables 2 and 3 show the comparison between patients with general adverse events versus adverse effects associated with MMF. Among patients with general adverse events, serum aspartate aminotransferase levels were higher. There were no significant differences in other variables.

At the time of LLDAS achievement, patients in the no-AE and AE groups had a mean daily GC dose of 7.0 (6.0–7.0) mg and 7.0 (7.0–7.13) mg (*P* = 0.27), respectively. The proportion of patients using MMF in the two groups was 93% and 0% (not applicable), respectively. The proportion of patients using tacrolimus in the two groups was 46% and 20% (*P* = 0.17), respectively. There was one patient treated with azathioprine in the no-AE group and one patient treated with belimumab in the AE group.

Figure 1(b) shows the Kaplan-Meier curve for the overall MMF discontinuation rate.  Figure 1(c) shows the Kaplan-Meier curve for the MMF discontinuation rate due to adverse events. Fifty-five percent of adverse events occurred in the first 2 months after the start of MMF therapy, and 79% occurred in the first 6 months.  Figure 1(d) shows the MMF dose at the last observation. The median MMF dose at the last observation was 1,000 (1,000–1,500) mg.

Seven severe infections occurred, consisting of two cases of varicella-zoster virus (VZV) meningitis, and one case each of disseminated VZV, urosepsis, osteomyelitis of the mandible, necrotizing fasciitis, and multiple subcutaneous abscesses. The GC dose in each patient at the time of infection was 60 mg, 55 mg, 55 mg, 35 mg, 30 mg, 28 mg, and 14 mg daily, respectively. One patient with disseminated VZV died during the observation period. There were four mild infections consisting of upper respiratory infections and mycobacterial dermatitis. Supplemental Table 4 shows the details of immunosuppressive therapies and outcomes in each patient.

We examined serum trough MPA concentrations (Table 2) in our patients' surplus frozen serum samples, which were stored as a general practice. All 11 patients received MMF as induction therapy. Reliable trough levels could only be measured in 11 samples. It was not possible to measure trough levels in the other samples because the timing of blood collection and oral MMF administration was uncertain. Two patients who had irreversible brain damage due to viral meningitis had MPA concentrations of 8.3 and 6.3 *μ*g/mL, respectively.

## 4. Discussion

We analyzed 119 patients with SLE treated with MMF. The overall discontinuation rate was 42.4%, the adverse event-related discontinuation rate was 34.0%, and there were 29 adverse events. Although the most reason for discontinuation was 11 infection, we considered that it was also influenced by the high median GC of 20 (10–40) mg/dL. These findings correspond to real-world safety data for MMF in Japanese patients with SLE.

The effectiveness of MMF for patients with SLE was also revealed in our study. The LLDAS achievement rate was 57%. Several patients were still tapering from GCs at the last observation, so the prevalence of LLDAS might have been higher if the observation period was extended. However, the interpretation of these results was limited due to the lack of standard therapeutic regimens and uniform follow-up duration. The no-AE group had a significantly higher LLDAS achievement rate, which might indicate the effectiveness of MMF in Japanese patients with SLE.

We analyzed the safety of MMF in Japanese patients with SLE. Life-threatening adverse events included seven severe infections. In order to use MMF safely, we consider it necessary to analyze factors that might predict adverse events. The analysis showed that lower hemoglobin, higher BUN, and use of other immunosuppressants are associated with adverse events. However, anemia was not associated with serum MPA levels in a previous report [18]. These results may not reflect renal dysfunction, because only BUN was associated with adverse events, not creatinine, and eGFR. Unfortunately, these results may include confounding factors because hemoglobin, BUN, and use of other immunosuppressants are correlated with each other and other factors. Focusing on the trends of higher anti-DNA antibodies, proteinuria, SLE disease activity index, and lower complement concentrations instead of anemia and BUN suggests that the AE group may have more active SLE. However, no statistical differences were observed; these findings should be verified in a larger study. We found MPA levels of 8.3 and 6.3 *μ*g/mL, respectively, in two patients with VZV meningitis. MMF was associated with increased susceptibility to VZV infection in previous studies on kidney transplantation [19, 20] and SLE [21, 22]. There is a Japanese case report of SLE and fatal VZV infection [23]. Physicians should be aware of the risk of viral infections such as VZV in patients with SLE taking MMF.

In this study, the median dose of MMF at the last observation was 1,000 mg, reflecting physicians' real-world choices for SLE maintenance therapy in Japan. EULAR recommends 3,000 mg of MMF for induction therapy and 2,000 mg of MMF for maintenance remission therapy in non-Asian patients with SLE and 2,000 mg of MMF for induction therapy in Asian patients with SLE. Because GCs interfere with MPA bioavailability, patients had higher MPA concentrations while being tapered off GCs after induction therapy than during induction therapy [24].

The usefulness of therapeutic drug monitoring for MPA in patients with SLE is controversial. Several studies on SLE reported that the concentration of MPA is associated with therapeutic effect and adverse events [25–27], only therapeutic effects [28–36], or neither [13]. Higher MPA concentration is associated with effectiveness in lupus nephritis during therapy to induce remission. Actual mean daily MMF doses were appropriate 1,500–2,000 mg [13, 27–29, 31, 32]. In these studies, the mean predose MPA concentration was between 1.7 and 2.5 *μ*g/mL. Higher MPA concentration during maintenance therapy is associated with favorable outcomes [13, 25–27, 31, 33–36]. The mean daily MMF dose was 1,900–2,000 mg, and the mean predose MPA concentration was between 1.7 and 4.2 *μ*g/mL. Trough levels did not correspond to the area under the blood concentration-time curve in patients with SLE [13, 25]. We could not conclude that therapeutic drug monitoring was useful. Further evaluation is needed.

A noteworthy point of our study was that MPA levels were measured retrospectively. If we had confirmed high serum MPA levels during treatment, we may have reduced the dose of MMF. Due to the difficulties in reliably measuring trough levels in a retrospective study, serum trough MPA levels could only be examined in 11 patients. This was the major limitation of our study.

## 5. Conclusion

We evaluated the rate of MMF continuation and reasons for MMF discontinuation in Japanese patients with SLE. High serum MPA levels may be associated with severe infections in Japanese patients with SLE. Although MMF may be effective in Japanese patients with SLE, physicians should pay attention to infections in patients with high MPA concentrations.

## Figures and Tables

**Table 1:** Characteristics of patients in the AE and no-AE groups.

	Overall	No-AE group	AE group	*P* value
	*N* = 119	*n* = 90	*n* = 29	
Age at start of MMF therapy, median (IQR)	38 (31–46)	39 (31–46)	33 (29–45)	0.20
Female sex, *n* (%)	101 (85)	74 (82)	27 (93)	0.23
Body weight (kg), median (IQR)	55 (48–63)	57 (48–64)	54 (46–62)	0.23
Duration of SLE, months, median (IQR)	138 (39–242)	133 (40–227)	160 (23–262)	0.68
MMF started as induction therapy, *n* (%)	96 (81)	72 (81)	24 (83)	1.00
Malar rash, *n* (%)	62 (52)	51 (57)	11 (38)	0.09
Discoid rash, *n* (%)	23 (20)	16 (18)	7 (24)	0.59
Photosensitivity, *n* (%)	31 (26)	21 (24)	10 (35)	0.15
Oral ulcers, *n* (%)	31 (27)	20 (23)	11 (38)	0.33
Arthritis, *n* (%)	75 (64)	54 (61)	21 (72)	0.28
Serositis, *n* (%)	28 (24)	23 (26)	5 (17)	0.45
Renal disorder, *n* (%)	81 (68)	62 (69)	19 (66)	0.82
Neurologic disorder, *n* (%)	9 (8)	6 (7)	3 (10)	0.69
Hematologic disorder, *n* (%)	89 (75)	68 (76)	21 (72)	0.8
Immunologic disorder, *n* (%)	118 (99)	89 (99)	29 (100)	1.00
Antinuclear antibody, *n* (%)	119 (100)	90 (100)	29 (100)	N/A
SLEDAI at start of MMF therapy, median (IQR)	6 (4–10)	6 (4–10)	8 (4–13)	0.14
Number of SLE flare-ups, median (IQR)	1 (1–3)	2 (1–2)	1 (0–3)	0.30
WBC count (/*μ*L), median (IQR)	6,600 (4,500–7,900)	6,100 (4,400–7,600)	7,000 (5,800–9,500)	0.07
Lymphocytes (/*μ*L), median (IQR)	1,001 (598–1,335)	925 (544–1,326)	1,039 (619–1,404)	0.41
Hemoglobin (g/dL), median (IQR)	11.8 (10.6–13.1)	12.1 (11.1–13.2)	11.2 (10–12.3)	0.014^∗^
Platelets (10^4^/*μ*L), median (IQR)	23.3 (18.6–28.8)	23.6 (19.3–28)	22.4 (17.8–30.7)	0.91
AST (U/L), median (IQR)	19 (15–24)	18 (15–24)	19 (15–25)	0.67
ALT (U/L), median (IQR)	18 (12–28)	16 (11–25)	25 (15–34)	0.011
Albumin (g/dL), median (IQR)	3.5 (3–3.9)	3.6 (3.1–3.9)	3.2 (2.6–3.8)	0.06
BUN (mg/dL), median (IQR)	15 (11–20)	14 (10–19)	18 (14–27)	0.006^∗∗^
Creatinine (mg/dL), median (IQR)	0.62 (0.49–0.83)	0.62 (0.49–0.78)	0.57 (0.48–0.99)	0.48
eGFR (mL/min/1.73 m^2^), median (IQR)	91 (67–115)	92 (70–115)	89 (46–124)	0.63
CH50 (U/mL), median (IQR)	28 (20–38)	29 (21–39)	25 (16–37)	0.33
C3 (mg/dL), median (IQR)	66 (48–84)	69 (52–85)	61 (44–83)	0.38
C4 (mg/dL), median (IQR)	11 (7–18)	12 (7–19)	10 (5–17)	0.31
IgG (mg/dL), median (IQR)	1,220 (914–1,455)	1,243 (943–1,431)	1,151 (597–1,718)	0.59
Anti-DNA antibody (RIA) (IU/mL), median (IQR)	11 (0–62)	7 (0–50)	30 (5–188)	0.053
Anti-U1-RNP antibody, positivity, *n* (%)	43 (36)	34 (38)	9 (31)	0.52
Anti-Sm antibody, positivity, *n* (%)	16 (14)	14 (16)	2 (7)	0.35
Anti-CL antibody, positivity, *n* (%)	26 (22)	22 (25)	4 (14)	0.30
Anti-CL*β*2GP1 antibody, positivity, *n* (%)	13 (11)	9 (10)	4 (14)	1.00
Lupus anticoagulant, median (IQR)	0.9 (0.9–1)	1 (0.9–1)	0.9 (0.8–1)	0.51
Proteinuria (g/day), median (IQR)	0.6 (0–2.3)	0.5 (0–2.1)	0.7 (0–2.5)	0.46
Hematuria, positivity, *n* (%)	38 (33)	28 (33)	10 (35)	1.00
GC dose at start of MMF therapy (mg/day), median (IQR)	20 (10–40)	19 (10–36)	28 (12–55)	0.07
Overall maximum GC dose (mg/day), median (IQR)	50 (40–60)	50 (40–60)	50 (40–60)	0.93
LLDAS at last observation, *n* (%)	67 (57)	57 (64)	10 (35)	0.009^∗∗^

AE: adverse event; MMF: mycophenolate mofetil; IQR: interquartile range; SLE: systemic lupus erythematosus; SLEDAI: SLE disease activity index; WBC: white blood cell; AST: aspartate aminotransferase; ALT: alanine aminotransferase; BUN: blood urea nitrogen; eGFR: estimated glomerular filtration rate; IgG: immunoglobulin G; DNA: deoxyribonucleic acid; RIA: radioimmunoassay; U1RNP: U1-ribonucleoprotein; Sm: Smith; CL: cardiolipin; CL*β* 2GP1: cardiolipin *β*2-glycoprotein I; GC: glucocorticoid; LLDAS: lupus low disease activity state. ^∗^*P* < 0.05. ^∗∗^*P* < 0.01.

**Table 2:** Characteristics of patients with measured MPA trough levels.

Patient	Age at start of MMF therapy (years)	Sex	Duration of MMF therapy (month)	MMF trough level (*μ*g/mL)	MMF dose (mg/day)	SLEDAI at start of MMF therapy	Outcome of MMF	Adverse event	Achievement of LLDAS
1	32	Male	5	8.5	2,000	14	Withdrawal	Renal dysfunction	No
2	43	Female	16	8.3	2,000	12	Withdrawal	Infection (VZV meningitis)	Yes
3	38	Female	1	7.3	1,500	18	Dose decrease		No
4	43	Female	2	6.7	2,000	14	Withdrawal	Cytopenia	No
5	46	Male	20	6.6	2,000	6	Dose decrease		Yes
6	32	Female	4	6.3	2,000	14	Withdrawal	Infection (VZV meningitis)	No
7	32	Female	2	5.3	1,500	16	Dose decrease		No
8	18	Female	12	4.7	2,000	12	Dose decrease		No
9	38	Female	11	4	1,000	9	Continue		No
10	23	Female	3	3.5	2,000	8	Withdrawal	Alopecia	Yes
11	23	Female	1	3.1	2,000	20	Withdrawal	Cytopenia	No

MPA: mycophenolate acid; MMF: mycophenolate mofetil; SLEDAI: systemic lupus erythematosus disease activity index; LLDAS: lupus low disease activity state.

## Data Availability

The data used to support the findings of this study are available from the corresponding author upon request.

## Supplementary Materials

Supplementary Materials Supplementary Table 1: characteristics of patients taking MMF as induction versus maintenance therapy. Supplementary Table 2: characteristics of patients with had general AEs versus adverse effects associated with MMF. Supplementary Table 3: patient characteristics by reason for MMF discontinuation. Supplementary Table 4: characteristics of patients who discontinued MMF due to severe infection.
