# Supplementary material for: Real-World Experience of Safety of Mycophenolate Mofetil in 119 Japanese Patients with Systemic Lupus Erythematosus: A Retrospective Single-Center Study
Source: Biomed Res Int. 2021 Jan 23;2021:8630596. doi: 10.1155/2021/8630596 (PMC7850850; doi:10.1155/2021/8630596)
Supplement: Supplementary Materials — Supplementary Table 1: characteristics of patients taking MMF as induction versus maintenance therapy. Supplementary Table 2: characteristics of patients with had general AEs versus adverse effects associated with MMF. Supplementary Table 3: patient characteristics by reason for MMF discontinuation. Supplementary Table 4: characteristics of patients who discontinued MMF due to severe infection. [file 8630596.f1.docx]

Supplementary Table 1. Characteristics of patients taking MMF as induction versus maintenance therapy

|  | Induction group | Maintenance group | *P* value |
| --- | --- | --- | --- |
|  | n = 96 | n = 22 |  |
| Age at start of MMF therapy, median (IQR) | 38 (31–46) | 39 (29–45) | 0.19 |
| Female sex, n (%) | 82 (85) | 19 (86) | 1.00 |
| Body weight, kg, median (IQR) | 55 (48–62) | 57 (46–67) | 0.62 |
| Duration of SLE, months, median (IQR) | 152 (45–243) | 60 (15–187) | 0.07 |
| Malar rash, n (%) | 50 (52) | 12 (55) | 1.00 |
| Discoid rash, n (%) | 21 (22) | 1 (5) | 0.12 |
| Photosensitivity, n (%) | 26 (27) | 4 (18) | 0.43 |
| Oral ulcers, n (%) | 26 (27) | 4 (18) | 0.43 |
| Arthritis, n (%) | 59 (61) | 15 (68) | 0.63 |
| Serositis, n (%) | 23 (24) | 5 (23) | 1.00 |
| Renal disorder, n (%) | 64 (67) | 16 (73) | 0.80 |
| Neurologic disorder, n (%) | 6 (6) | 3 (14) | 0.37 |
| Hematologic disorder, n (%) | 71 (74) | 17 (77) | 1.00 |
| Immunologic disorder, n (%) | 94 (98) | 22 (100) | 1.00 |
| Antinuclear antibody, n (%) | 95 (99) | 21 (95) | N/A |
| SLEDAI at start of MMF therapy, median (IQR) | 6 (4–11) | 6 (4–9) | 0.45 |
| Number of SLE flare-ups, median (IQR) | 2 (1–3) | 1 (0–2) | 0.24 |
| WBC count, /μL, median (IQR) | 6750 (4400–8350) | 5800 (4950–7000) | 0.25 |
| Lymphocytes, /μL, median (IQR) | 1004 (620–1297) | 871 (537–1399) | 0.67 |
| Hemoglobin, g/dL, median (IQR) | 12 (11–13) | 12 (10–14) | 0.65 |
| Platelets, 10^4^/μL, median (IQR) | 24 (19–29) | 22 (19–25) | 0.39 |
| AST, U/L, median (IQR) | 18 (15–24) | 20 (15–28) | 0.79 |
| ALT, U/L, median (IQR) | 18 (12–28) | 15 (11–28) | 0.50 |
| Albumin, g/dL, median (IQR) | 4 (3–4) | 4 (3–4) | 0.33 |
| BUN, mg/dL, median (IQR) | 15 (11–20) | 14 (11–18) | 0.37 |
| Creatinine, mg/dL, median (IQR) | 0.61 (0.48–0.89) | 0.64 (0.52–0.79) | 0.99 |
| eGFR, mL/min/1.73m^2^, median (IQR) | 91 (66–118) | 92 (73–112) | 0.98 |
| CH50, U/mL, median (IQR) | 26 (18–36) | 34 (27–43) | 0.01* |
| C3, mg/dL, median (IQR) | 64 (46–81) | 74 (60–91) | 0.11 |
| C4, mg/dL, median (IQR) | 11 (6–17) | 14 (8–19) | 0.22 |
| IgG, mg/dL, median (IQR) | 1235 (928–1475) | 1163 (789–1434) | 0.57 |
| Anti-DNA antibody (RIA), IU/mL, median (IQR) | 11 (0–78) | 6 (0–43) | 0.02* |
| Anti-U1-RNP antibody positivity, n (%) | 33 (34) | 10 (45) | 0.32 |
| Anti-Sm antibody positivity, n (%) | 12 (13) | 5 (23) | 0.19 |
| Anti-CL antibody positivity, n (%) | 22 (23) | 4 (18) | 1.00 |
| Anti-CLβ2GP1 antibody positivity, n (%) | 12 (13) | 1 (5) | 0.46 |
| Lupus anticoagulant, median (IQR) | 0.9 (0.9–1) | 0.9 (0.9–1) | 0.60 |
| Proteinuria, g/day, median (IQR) | 0.7 (0–2.3) | 0.6 (0–1.8) | 0.71 |
| Hematuria, positivity, n (%) | 33 (34) | 5 (23) | 0.32 |
| GC dose at start of MMF therapy, mg/day, median (IQR) | 21 (10–50) | 20 (7–28) | 0.04* |
| Overall maximum GC dose, mg/day, median (IQR) | 50 (40–60) | 50 (35–60) | 0.79 |
| LLDAS at last observation, n (%) | 55 (57) | 11 (50) | 0.81 |

MMF, mycophenolate mofetil; IQR, interquartile range; SLE, systemic lupus erythematosus; SLEDAI, SLE disease activity index; WBC, white blood cell; AST, aspartate aminotransferase; ALT, alanine aminotransferase; BUN, blood urea nitrogen; eGFR, estimated glomerular filtration rate; IgG, immunoglobulin G; DNA, deoxyribonucleic acid; RIA, radioimmunoassay; U1RNP, U1-ribonucleoprotein; Sm, Smith; CL, cardiolipin; CLβ2GP1, cardiolipin β2-glycoprotein I; GC, glucocorticoid; LLDAS, lupus low disease activity state.

**P* < 0.05

Supplementary Table 2. Characteristics of patients with had general AEs versus adverse effects associated with MMF

|  | General AE | MMF-associated adverse effect | *P* value |
| --- | --- | --- | --- |
|  | n = 14 | n = 15 |  |
| Age at start of MMF therapy, median (IQR) | 34 (29–44) | 32 (29–51) | 0.98 |
| Female sex, n (%) | 13 (93) | 14 (93) | 1.00 |
| Body weight, kg, median (IQR) | 54 (46–60) | 53 (46–63) | 0.91 |
| Duration of SLE, months, median (IQR) | 202 (2–268) | 160 (32–239) | 0.81 |
| MMF started as induction therapy, n (%) | 11 (79) | 13 (87) | 0.65 |
| Malar rash, n (%) | 5 (36) | 6 (40) | 1.00 |
| Discoid rash, n (%) | 3 (21) | 4 (27) | 1.00 |
| Photosensitivity, n (%) | 4 (29) | 6 (40) | 0.70 |
| Oral ulcers, n (%) | 3 (21) | 8 (53) | 0.13 |
| Arthritis, n (%) | 10 (71) | 11 (73) | 1.00 |
| Serositis, n (%) | 3 (21) | 2 (13) | 0.65 |
| Renal disorder, n (%) | 11 (79) | 8 (53) | 0.25 |
| Neurologic disorder, n (%) | 2 (14) | 1 (7) | 0.60 |
| Hematologic disorder, n (%) | 11 (79) | 10 (67) | 0.68 |
| Immunologic disorder, n (%) | 14 (100) | 15 (100) | N/A |
| Antinuclear antibody, n (%) | 14 (100) | 15 (100) | N/A |
| SLEDAI at start of MMF therapy, median (IQR) | 8 (4–13) | 8 (4–14) | 0.91 |
| Number of SLE flare-ups, median (IQR) | 1 (0–3) | 1 (0–3) | 0.75 |
| WBC count, /μL, median (IQR) | 8600 (6350–9950) | 6500 (4500–7000) | 0.08 |
| Lymphocytes, /μL, median (IQR) | 950 (621–1232) | 1039 (619–2036) | 0.50 |
| Hemoglobin, g/dL, median (IQR) | 12 (10–13) | 11 (10–12) | 0.27 |
| Platelets, 10^4^/μL, median (IQR) | 21 (18–26) | 25 (15–31) | 0.53 |
| AST, U/L, median (IQR) | 24 (18–35) | 17 (14–22) | 0.02* |
| ALT, U/L, median (IQR) | 34 (18–59) | 19 (14–29) | 0.06 |
| Albumin, g/dL, median (IQR) | 3 (2–4) | 4 (3–4) | 0.26 |
| BUN, mg/dL, median (IQR) | 17 (13–30) | 19 (14–26) | 0.62 |
| Creatinine, mg/dL, median (IQR) | 0.7 (0.49–1.11) | 0.55 (0.47–0.99) | 0.65 |
| eGFR, mL/min/1.73m^2^, median (IQR) | 83 (46–118) | 93 (46–130) | 0.59 |
| CH50, U/mL, median (IQR) | 29 (20–40) | 21 (15–36) | 0.21 |
| C3, mg/dL, median (IQR) | 69 (56–95) | 51 (38–70) | 0.11 |
| C4, mg/dL, median (IQR) | 10 (6–20) | 9 (5–14) | 0.40 |
| IgG, mg/dL, median (IQR) | 936 (686–1706) | 1151 (556–1740) | 0.72 |
| Anti-DNA antibody (RIA), IU/mL, median (IQR) | 5 (0–82) | 50 (14–200) | 0.052 |
| Anti-U1-RNP antibody positivity, n (%) | 6 (43) | 3 (20) | 0.25 |
| Anti-Sm antibody positivity, n (%) | 2 (14) | 1 (7) | 0.60 |
| Anti-CL antibody positivity, n (%) | 3 (21) | 1 (7) | 0.33 |
| Anti-CLβ2GP1 antibody positivity, n (%) | 2 (14) | 2 (13) | 1.00 |
| Lupus anticoagulant, median (IQR) | 0.9 (0.6–1) | 0.9 (0.8–1) | 0.56 |
| Proteinuria, g/day, median (IQR) | 0.9 (0–2.6) | 0.4 (0–2.7) | 0.57 |
| Hematuria, positivity, n (%) | 2 (14) | 8 (53) | 0.050 |
| GC dose at start of MMF therapy, mg/day, median (IQR) | 33 (19–55) | 25 (10–50) | 0.33 |
| Overall maximum GC dose, mg/day, median (IQR) | 55 (43–60) | 50 (40–60) | 0.56 |
| LLDAS at last observation, n (%) | 3 (21) | 7 (47) | 0.25 |

AE, adverse event; MMF, mycophenolate mofetil; IQR, interquartile range; SLE, systemic lupus erythematosus; SLEDAI, SLE disease activity index; WBC, white blood cell; AST, aspartate aminotransferase; ALT, alanine aminotransferase; BUN, blood urea nitrogen; eGFR, estimated glomerular filtration rate; IgG, immunoglobulin G; DNA, deoxyribonucleic acid; RIA, radioimmunoassay; U1RNP, U1-ribonucleoprotein; Sm, Smith; CL, cardiolipin; CLβ 2GP1, cardiolipin β2-glycoprotein I; GC, glucocorticoid; LLDAS, lupus low disease activity state.

**P* < 0.05

Supplementary Table 3. Patient characteristics by reason for MMF discontinuation

|  | Infection | Nausea or  diarrhea | SLE  exacerbation | Cytopenia | Renal  dysfunction | Liver  dysfunction | Alopecia | Rash |
| --- | --- | --- | --- | --- | --- | --- | --- | --- |
|  | n = 11 | n = 9 | n = 3 | n = 2 | n = 1 | n = 1 | n = 1 | n = 1 |
| Age at start of MMF therapy, median (IQR) | 35 (32–43) | 32 (30–54) | 20 (17–46) | 23-33 | 32 | 52 | 23 | 38 |
| Female sex, n (%) | 10 (91) | 9 (100) | 3 (100) | 2 (100) | 0 (0) | 1 (100) | 1 (100) | 1 (100) |
| Body weight, kg, median (IQR) | 54 (42–59) | 47 (43–65) | 57 (48–63) | 53-61 | 56 | 63 | 53 | 47 |
| Duration of SLE, months, median (IQR) | 258 (53–272) | 160 (23–290) | 2 (2–263) | 1-65 | 237 | 239 | 102 | 171 |
| MMF started as induction therapy, n (%) | 3 (27) | 2 (22) | 0 (0) | 0 (0) | 0 (0) | 0 (0) | 0 (0) | 0 (0) |
| Malar rash, n (%) | 3 (27) | 3 (33) | 2 (67) | 2 (100) | 1 (100) | 0 (0) | 0 (0) | 0 (0) |
| Discoid rash, n (%) | 2 (18) | 1 (11) | 1 (33) | 2 (100) | 1 (100) | 0 (0) | 0 (0) | 0 (0) |
| Photosensitivity, n (%) | 4 (36) | 3 (33) | 0 (0) | 2 (100) | 1 (100) | 0 (0) | 0 (0) | 0 (0) |
| Oral ulcers, n (%) | 1 (9) | 4 (44) | 2 (67) | 2 (100) | 0 (0) | 1 (100) | 0 (0) | 1 (100) |
| Arthritis, n (%) | 7 (64) | 7 (78) | 3 (100) | 2 (100) | 0 (0) | 0 (0) | 1 (100) | 1 (100) |
| Serositis, n (%) | 1 (9) | 0 (0) | 2 (67) | 0 (0) | 1 (100) | 1 (100) | 0 (0) | 0 (0) |
| Renal disorder, n (%) | 9 (82) | 4 (44) | 2 (67) | 2 (100) | 1 (100) | 0 (0) | 1 (100) | 0 (0) |
| Neurologic disorder, n (%) | 2 (18) | 0 (0) | 0 (0) | 1 (50) | 0 (0) | 0 (0) | 0 (0) | 0 (0) |
| Hematologic disorder, n (%) | 8 (73) | 6 (67) | 3 (100) | 2 (100) | 1 (100) | 0 (0) | 1 (100) | 0 (0) |
| Immunologic disorder, n (%) | 11 (100) | 9 (100) | 3 (100) | 2 (100) | 1 (100) | 1 (100) | 1 (100) | 1 (100) |
| Antinuclear antibody, n (%) | 11 (100) | 9 (100) | 3 (100) | 2 (100) | 1 (100) | 1 (100) | 1 (100) | 1 (100) |
| SLEDAI at start of MMF therapy, median (IQR) | 6 (4–12) | 6 (3–12) | 9 (6–16) | 14–17 | 14 | 8 | 8 | 4 |
| Number of SLE flare-ups, median (IQR) | 1 (0–3) | 1 (0–3) | 0 (0–5) | 0-1 | 1 | 1 | 3 | 2 |
| WBC count, /μL, median (IQR) | 9200 (6625–10350) | 5900 (3800–7250) | 6900 (5800–8600) | 4500–5750 | 6700 | 11600 | 6500 | 7000 |
| Lymphocytes, /μL, median (IQR) | 901 (525–1051) | 963 (567–2070) | 1304 (1160–2537) | 761–906 | 1,039 | 1218 | 2308 | 1519 |
| Hemoglobin, g/dL, median (IQR) | 12 (9–13) | 11 (10–11) | 13 (11–13) | 44115 | 15 | 9 | 13 | 11 |
| Platelets, 10^4^/μL, median (IQR) | 21 (18–27) | 21 (14–31) | 23 (1–29) | 25–27 | 26 | 40 | 18 | 36 |
| AST, U/L, median (IQR) | 24 (19–49) | 16 (12–26) | 24 (15–31) | 14–18 | 20 | 17 | 15 | 17 |
| ALT, U/L, median (IQR) | 38 (19–63) | 19 (13–31) | 34 (10–54) | 44122 | 69 | 22 | 15 | 18 |
| Albumin, g/dL, median (IQR) | 3 (2–3) | 4 (3–4) | 4 (3–4) | 43864 | 2 | 4 | 3 | 4 |
| BUN, mg/dL, median (IQR) | 16 (11–31) | 19 (15–25) | 17 (13–30) | 21–30 | 26 | 11 | 8 | 14 |
| Creatinine, mg/dL, median (IQR) | 0.74 (0.49–1) | 0.54 (0.43–1.05) | 0.52 (0.43–1.78) | 0.48–0.79 | 0.99 | 0.55 | 0.47 | 0.57 |
| eGFR, mL/min/1.73m^2^, median (IQR) | 82 (49–112) | 106 (43–130) | 124 (25–160) | 44–87 | 73 | 89 | 133 | 93 |
| CH50, U/mL, median (IQR) | 30 (21–41) | 21 (15–28) | 26 (16–30) | 44179 | 0 | 36 | 37 | 36 |
| C3, mg/dL, median (IQR) | 70 (58–96) | 47 (39–61) | 68 (50–95) | 33–44 | 19 | 102 | 103 | 65 |
| C4, mg/dL, median (IQR) | 11 (6–26) | 9 (4–11) | 10 (3–10) | 44019 | 0 | 15 | 19 | 13 |
| IgG, mg/dL, median (IQR) | 936 (681–1456) | 1151 (515–2009) | 1791 (598–2411) | 1505–1616 | 1753 | 970 | 418 | 596 |
| Anti-DNA antibody (RIA), IU/mL, median (IQR) | 6 (0–140) | 50 (34–163) | 0 (0-44) | 200–200 | 200 | 12 | 0 | 18 |
| Anti-U1-RNP antibody positivity, n (%) | 5 (45) | 1 (11) | 1 (33) | 0 (0) | 1 (100) | 0 (0) | 1 (100) | 0 (0) |
| Anti-Sm antibody positivity, n (%) | 1 (9) | 0 (0) | 1 (33) | 0 (0) | 1 (100) | 0 (0) | 0 (0) | 0 (0) |
| Anti-CL antibody positivity, n (%) | 2 (18) | 1 (11) | 1 (33) | 0 (0) | 0 (0) | 0 (0) | 0 (0) | 0 (0) |
| Anti-CLβ2GP1 antibody positivity, n (%) | 1 (9) | 0 (0) | 1 (33) | 0 (0) | 1 (100) | 1 (100) | 0 (0) | 0 (0) |
| Lupus anticoagulant, median (IQR) | 0.9 (0–0.9) | 0.9 (0.9–1.15) | 1 (0.9–1.5) | 0.8–0.8 | 0.9 | 0.8 | 0.9 | 1 |
| Proteinuria, g/day, median (IQR) | 1.02 (0–2.48) | 0.35 (0–6.08) | 0.7 (0–3.1) | 0–1.8 | 1.6 | 0 | 2.4 | 0 |
| Hematuria, positivity, n (%) | 2 (18) | 4 (44) | 0 (0) | 1 (50) | 1 (100) | 1 (100) | 1 (100) | 0 (0) |
| GC dose at start of MMF therapy, mg/day, median (IQR) | 30 (20–55) | 10 (10–39) | 50 (8–55) | 40–53 | 65 | 8 | 30 | 15 |
| Overall maximum GC dose, mg/day, median (IQR) | 55 (35–60) | 50 (40–58) | 55 (50–60) | 40–53 | 65 | 20 | 40 | 50 |
| LLDAS at last observation, n (%) | 1 (9) | 4 (44) | 2 (67) | 0 (0) | 0 (0) | 1 (100) | 1 (100) | 1 (100) |

MMF, mycophenolate mofetil; IQR, interquartile range; SLE, systemic lupus erythematosus; SLEDAI, SLE disease activity index; WBC, white blood cell; AST, aspartate aminotransferase; ALT, alanine aminotransferase; BUN, blood urea nitrogen; eGFR, estimated glomerular filtration rate; IgG, immunoglobulin G; DNA, deoxyribonucleic acid; RIA, radioimmunoassay; U1RNP, U1-ribonucleoprotein; Sm, Smith; CL, cardiolipin; CLβ 2GP1, cardiolipin β2-glycoprotein I; GC, glucocorticoid; LLDAS, lupus low disease activity state.

Supplementary Table 4. Characteristics of patients who discontinued MMF due to severe infection

| Patient | Infection | Age, years | Sex | GC dose at the start of MMF therapy (mg/day) | Use of other ISs | Therapy type | Outcome |
| --- | --- | --- | --- | --- | --- | --- | --- |
| 1 | VZV meningitis | 43 | Female | 30 | Tacrolimus | Maintenance | Survived |
| 2 | VZV meningitis | 32 | Female | 55 | None | Induction | Survived |
| 3 | Disseminated VZV | 33 | Female | 55 | None | Induction | Died |
| 4 | Urosepsis | 29 | Female | 28 | None | Maintenance | Survived |
| 5 | Osteomyelitis of the mandible | 58 | Female | 60 | None | Induction | Survived |
| 6 | Necrotizing fasciitis | 29 | Female | 14 | None | Induction | Survived |
| 7 | Multiple subcutaneous abscesses | 42 | Female | 35 | None | Induction | Survived |
| 8 | Septic arthritis | 42 | Male | 20 | None | Maintenance | Survived |
| 9 | Cytomegalovirus infection | 32 | Female | 60 | None | Induction | Survived |
| 10 | Mycobacterial dermatitis | 35 | Female | 14 | None | Induction | Survived |
| 11 | Upper respiratory infection | 46 | Female | 23 | Tacrolimus | Induction | Survived |

MMF, mycophenolate mofetil; GC, glucocorticoid; IS, immunosuppressant; VZV, varicella-zoster virus.
